# Predictive value of the Adult Comorbidity Evaluation 27 on adverse surgical outcomes and survival in elderly with advanced epithelial ovarian cancer undergoing cytoreductive surgery

**DOI:** 10.1186/s40001-024-01666-1

**Published:** 2024-03-17

**Authors:** Mengna Zhao, Yang Gao, Junyuan Yang, Hao He, Min Su, Shimeng Wan, Xiaoye Feng, Hua Wang, Hongbing Cai

**Affiliations:** Department of Gynecological Oncology, Hubei Clinical Cancer Study Center, Hubei Key Laboratory of Tumor Biological Behaviors, Zhongnan Hospital of Wuhan University, No. 169 East Lake Road, Wuhan, 430071 People’s Republic of China

**Keywords:** ACE-27, Ovarian cancer, Surgery, Comorbidity, Elderly

## Abstract

**Objective:**

We aimed to evaluate the ability of Adult Comorbidity Evaluation 27 (ACE-27) to predict perioperative outcomes and survival in elderly women with advanced epithelial ovarian cancer (AEOC) undergoing cytoreductive surgery.

**Methods:**

We collected patients with AEOC in our hospital between January 1, 2012 and January 1, 2021. Patients younger than 65 years old or those with non-epithelial ovarian cancer were excluded. ACE-27 was applied retrospectively to assess comorbidities in the selected patients, who were then classified into two groups based on their ACE-27 scores: low ACE-27 score group (none to mild) and high ACE-27 score group (moderate to severe).

**Results:**

A total of 222 elderly women with AEOC were included, of whom 164 patients accepted debulking surgery. Among those who have undergone surgery, Clavien–Dindo grade III + perioperative complications or unintended intensive care unit (ICU) admission occurred more often in patients of high ACE-27 score group, with statistically significant difference (odds ratio [OR]: 4.21, 95% confidence interval [CI], 1.28–14.35,* p* = 0.018). Further stratified analyses by age, BMI, FIGO stage and pathology also prove that OS of patients graded severe was shorter than patients graded none to moderate in cohort of age < 70, BMI < 25 kg/m^2^, FIGO III stage and pathology of serous, respectively. Kaplan–Meier survival curves analyzed by log-rank test showed that the overall survival (OS) of patients with severe comorbidities were shorter than with none to moderate (HR 3.25, 95%CI 1.55–6.79, p = 0.002).

**Conclusions:**

Our findings demonstrate the ability of ACE-27 to predict grade III + perioperative complications or unintended ICU admission and survival in elderly patients with AEOC. This highlights the possibility for ACE-27 to play an instrumental role in identifying AEOC patients who are more susceptible to adverse surgical outcomes and have a poor survival rate and assisting in decisions regarding treatment.

**Supplementary Information:**

The online version contains supplementary material available at 10.1186/s40001-024-01666-1.

## Introduction

Ovarian cancer ranks among the leading causes of gynecological malignancy globally, contributing significantly to morbidity and mortality among women [1]. It was estimated that there were 313959 new cases and 207252 deaths in 2020, in which 110630 (27.1%) and 98376 (23.7%) happened to elderly patients aged 65 years and above [2]. Given the aging population and the increasing prevalence of ovarian cancer among older women, it is imperative to enhance the management of this patient group. Despite debulking surgery and platinum-based chemotherapy being the standard treatment for ovarian cancer, the proportion of elderly patients undergoing surgical intervention is lower compared to younger patients [3]. Moreover, this demographic group has often been underrepresented in cancer therapy clinical trials, leading to limited information available for clinical decision-making [4]. Various factors, including age, functional status, comorbidities, patient care goals, and financial resources, significantly influence the treatment decisions made by both clinicians and patients.

Surgery for ovarian cancer is known to be an evaluation procedure. Despite the Fagotti scoring system [5] and Suidan’s computed tomography (CT) scan system [6] for predicting residual disease, whether these scoring systems will benefit the elderly is unclear. The management of advanced epithelial ovarian cancer (AEOC) in elderly women continues to pose significant challenges due to severe perioperative complications.

The present study is designed to validate the ability of the Adult Comorbidity Evaluation 27 (ACE-27) to predict both short-term and long-term outcomes in AEOC patients 65 years of age or older undergoing debulking surgery at our facility. There are always co-existing disorders with ovarian cancer, known as comorbidities in the elderly [7]. The impact of comorbidities on ovarian cancer is not clear, as age is considered an independent predictive factor for poor prognosis. [8], and a validated comorbidity index for pre-operative assessment is still needed. Adult Comorbidity Evaluation 27 (ACE-27), evolved from Kaplan Feinstein Comorbidity Index (KFI) [9–11], was originally designed for cancer patients to assess comorbidities and lately validated in several types of cancers such as endometrial cancer and head and neck cancer [12, 13]. No studies have been obtained to assess the incidence and severity of comorbidities in ovarian cancer with ACE-27. The present study is therefore designed to validate the ability of ACE-27 to predict both short-term and long-term outcomes in AEOC patients 65 years of age or older undergoing debulking surgery at our facility.

## Patients and methods

With the surveillance of Medical Ethics Committee Zhongnan Hospital, Wuhan University, we collected consecutive patients aged 65 and over (65 +) from January 1, 2012 to January 1, 2022 with newly diagnosed FIGO stage IIB to IV epithelial ovarian cancer treated at our institution. The exclusion criteria were age younger than 65, non-epithelial ovarian cancer, or tumors of stage I to IIA.

Baseline information was gathered through our electronic medical record system, including age, date of diagnosis, Federation International of Gynecology and Obstetrics (FIGO) stage, histology, cancer antigen 125 (CA125) at diagnosis, albumin and body mass index (BMI). Physical status was assessed by Eastern Cooperative Oncology Group (ECOG).

The evaluation of patients for comorbidities was conducted by two independent investigators, who closely examined the medical records of each patient at the time of their surgery. ACE-27 index was used to grade the severity of organ system decompensation and its impact on prognosis (grade 0 = none, grade 1 = mild, grade 2 = moderate, grade 3 = severe)[14]. This methodology resulted in an overall comorbidity score, which was determined by the highest-ranked individual disease. However, the protocol dictated an exception: when two or more moderate conditions were present, but located in different organ systems, a final score indicating severe comorbidity was assigned [15]. With respect to patients with epithelial ovarian cancer, their specific condition was omitted from the scoring system. For statistical analysis, the study population was categorized into two groups based on ACE-27 grading: low ACE-27 score group (grade 0 to 1) and high ACE-27 score group (grade 2 to 3).

The study incorporates data pertinent to several surgical aspects: type of surgery (primary debulking surgery (PDS) or neoadjuvant chemotherapy followed by interval debulking surgery (NACT/IDS)), operation time, American Society of Anesthesiologist (ASA), Mayo Surgical Complexity Score (SCS) [16], ascites, perioperative complication, time to adjuvant chemotherapy, hospital length of stay (LOS). In terms of tumor status post-surgery, this was categorized using the residual disease classification: R0 represented no remaining tumor, R1 denoted residual disease ≤ 1 cm, whereas R2 implied remaining disease > 1 cm. Routine postoperative intensive care unit (ICU) admissions occurred on the first postoperative day, the unintended ICU admission included: stay in ICU for more than 2 days, or readmission to the ICU during the identical hospitalization. The Clavien–Dindo Classification system was used to assess perioperative complication severity [17], grade III to V perioperative complications were gathered as severe perioperative complications among them. Major complications in the study included pulmonary embolism, intestinal fistula, poor postoperative wound healing requiring another surgical intervention, severe pulmonary or abdominal infections requiring ICU management, acute heart failure, and acute respiratory failure. Overall survival (OS) was calculated from date of diagnosis to the date of death, with patients still alive censored on the date of last follow-up, December 31, 2022.

To conduct a comparative analysis between the low and high ACE-27 score groups, the Chi-square test or Fisher exact test was utilized for categorical variables, while Student’s t-test was applied for continuous variables. A multivariate logistic regression analysis was undertaken to determine if ACE-27 could be a predictor for adverse surgical outcomes, using severe perioperative complication or unintended ICU admission as the outcome variables. The Kaplan–Meier (K-M) method and log‐rank test were employed for survival analysis and to compare the difference in the survival distributions. All *p*-values presented are two-sided, and associations were considered to be significant if a *p* value < 0.05. R studio was used for logistic analysis and to plot graphs.

## Results

Of 349 initially qualified patients, 127 patients were eliminated for the following reasons: younger age at diagnosis than 65 (n = 31), FIGO stage of I to IIA (n = 15), non-epithelial ovarian cancer (n = 28), missing pathological information (n = 38) and metastatic ovarian cancer (n = 15), resulting in a final cohort of 222 women. 31.08% (n = 69) of the patients had an ACE-27 score of 0, 45.94% (n = 102) an ACE-27 score of 1, 13.51% (n = 30) an ACE-27 score of 2, and 9.46% (n = 21) an ACE-27 score of 3. The baseline characteristics are listed in Table 1. Within the cohort of patients aged 65 years or older, although the high ACE-27 score group appeared to have older median age compared to the low ACE-27 score group, statistical analysis revealed the age difference between the two groups was not significant (*p* = 0.06). The physical status evaluated by ECOG score in the high ACE-27 score group was worse (*p* = 0.001). Additionally, the ACE-27 score influenced surgical treatment decisions as there was a significantly higher rate of operation rejection among patients with high ACE-27 scores (*p* = 0.039).

**Table 1:** Baseline characteristics

Characteristic	ACE-27 low (0–1)	ACE-27 high (2–3)	*p* value
	n = 171	n = 51	
Age (median (IQR))	68 (66,72)	71 (66,75)	0.06
BMI (median (IQR))	22.86 (20.31,25.78)	22.27 (20.30,24.62)	0.314
ECOG			
0 n(%)	84 (49.12)	16 (31.37)	0.001
1 n(%)	65 (38.01)	17 (33.33)	
≥ 2 n(%)	22 (12.86)	18 (35.29)	
FIGO stage			
IIB n(%)	20 (11.69)	1 (1.960)	0.034*
III n(%)	101 (59.06)	37 (72.54)	
IV n(%)	37 (21.63)	7 (13.72)	
Histology			
Serous n(%)	140 (81.87)	33 (64.70)	0.001*
Mucinous n(%)	8 (4.678)	5 (9.803)	
Endometrioid n(%)	4 (2.339)	5 (9.803)	
Clear n(%)	1 (0.584)	4 (7.843)	
Mixed + others n(%)	18 (10.52)	4 (7.843)	
Albumin			
< 30 g/L n(%)	15 (8.771)	9 (17.64)	0.109
30 ~ 35 g/L n(%)	35 (20.46)	7 (13.72)	
> 35 g/L n(%)	78 (45.61)	19 (37.25)	
CA125			
< 600 n(%)	56 (32.74)	17 (33.33)	0.383
≥ 600 n(%)	91 (53.21)	20 (39.21)	
Treatment			
no-CRS n(%)	39 (22.80)	19 (37.25)	0.039
CRS n(%)	132 (77.19)	32 (62.74)	

ACE-27, Adult Comorbidity Evaluation 27; IQR, interquartile range; BMI, body mass index; ECOG, Eastern Cooperative Oncology Group; FIGO, Federation International of Gynecology and Obstetrics; CRS, cytoreductive surgery;

^*^ Fisher exact test

Table 2 outlines the comorbidity profile. As the most common disease in the elderly, hypertension accounted for 47.29% of the cohort, followed by diabetes mellitus (16.66%), angina/coronary artery disease (13.51%), respiratory system (8.10%), congestive heart failure (5.85%), solid tumor including melanoma (5.40%), venous disease (5.40%), arrhythmias (5.40%), hepatic (4.50%) successively. 38.74% of the aged patients (n = 86) in the study suffered two or more comorbidities.

**Table 2:** Detailed ACE-27 grading

Cogent comorbid	Grade1	Grade2	Grade3	SUM (%)
ailment	Mild decompensation	Moderate decompensation	Severe decompression	
Cardiovascular system				
Myocardial infarct	0	1	1	2 (0.90)
Angina/coronary artery disease	29	1	0	30 (13.51)
Congestive heart failure (CHF)	4	6	3	13 (5.85)
Arrhythmias	2	8	2	12 (5.40)
Hypertension	102	3	0	105 (47.29)
Venous disease	4	7	1	12 (5.40)
Peripheral arterial disease	1	0	1	2 (0.90)
Respiratory system	11	5	2	18 (8.10)
Gastrointestinal system				
Hepatic	8	1	1	10 (4.50)
Stomach/intestine	7	0	0	7 (3.15)
Pancreas	0	0	0	0 (0.00)
Renal system				
End-stage renal disease	3	0	0	3 (1.35)
Endocrine system (code the comorbid ailments with (*) in both the endocrine system and other organ systems if applicable)				
Diabetes mellitus	25	11	1	37 (16.66)
Neurological system				
Stroke	5	2	0	7 (3.15)
Dementia	0	1	0	1 (0.45)
Paralysis	0	0	0	0 (0.00)
Neuromuscular	1	0	0	1 (0.45)
Psychiatric	0	0	0	0 (0.00)
Rheumatologic (including rheumatoid arthritis, systemic lupus, mixed connective tissue disorder, polymyositis, rheumatic polymyositis)	4	1	0	5 (2.25)
Immunological system				
AIDS	0	0	0	0 (0.00)
Malignancy (excluding cutaneous basal cell Ca., cutaneous SCCA, carcinoma in situ and intraepithelial neoplasm)				
Solid tumor including melanoma	8	4	0	12 (5.40)
Leukemia or myeloma	0	0	0	0 (0.00)
Lymphoma	0	0	0	0 (0.00)
Substance abuse	0	0	0	0 (0.00)
Alcohol	0	0	0	0 (0.00)
Illicit drugs	0	0	0	0 (0.00)
Body weight obesity	–	0	–	0 (0.00)
Final grading				
ACE-27 n(%)	102 (45.94)	30 (13.51)	21 (9.46)	152 (68.47)

ACE-27, Adult Comorbidity Evaluation 27; AIDS, Acquired immunodeficiency syndrome

Surgery characteristics are listed in Table 3. Of 222 elderly women with ovarian cancer included in the study, 164 (73.87%) patients accepted cytoreductive surgery (CRS). Regardless of whether primary debulking surgery (PDS) or interval debulking surgery following neoadjuvant chemotherapy (IDS/NACT), the surgical types did not exhibit any consequential differences between the two groups (*p* = 0.778). The high ACE-27 score group experienced significantly shorter operation time than the low ACE-27 score group (median (interquartile range [IQR]): 3.8 h (2.7, 4.8) versus 4.6 h (3.5, 5.3), *p* = 0.016). There was a significant difference between the two groups with regard to adverse surgical outcomes, patients who scored higher were more likely to suffer from Clavien–Dindo III–V complications during the perioperative stage (6.82% versus 21.87%, *p* = 0.018) or to be admitted into the intensive care unit unexpectedly (25% versus 9.09%, *p* = 0.014). When taking the grade III + complication or unintended ICU admission as a composite indicator, the difference between the two groups is significant (12.12% versus 34.37%, *p* = 0.002). A subsequent investigation was conducted to assess the potential impact of certain comorbidities on grade III + perioperative complications or unintentional ICU admissions (Additional file 1: Table S1). The results demonstrated a significant association of two specific comorbidities in univariate logistic analysis: arrhythmias (odds ratio [OR]: 7.77, 95% confidence interval [CI], 1.61–41.64; *p* = 0.01) and congestive heart failure (OR: 17, 95%CI, 2.08–351.59, *p* = 0.016).

**Table 3:** Surgery related information

Characteristic	ACE-27 low (0–1)	ACE-27 high (2–3)	*p* value
	n = 132	n = 32	
Age (median (IQR))	68 (66,70)	67 (66,71)	0.534
Type of surgery			
PDS n(%)	100 (75.75)	25 (78.12)	0.778
NACT/IDS n(%)	31 (23.48)	7 (21.87)	
SCS			
≤ 3 n(%)	94 (71.21)	25 (78.12)	0.284
≥ 4 n(%)	33 (25.00)	5 (15.62)	
Operation time (median (IQR))	4.6 (3.5,5.3)	3.8 (2.7,4.8)	0.016
ASA			
I–II n(%)	97 (73.48)	20 (62.5)	0.218
III–IV n(%)	35 (26.51)	12 (37.5)	
Residual disease			
R0 n(%)	81 (61.36)	17 (53.12)	0.41*
R1 n(%)	29 (21.96)	8 (25.00)	
R2 n(%)	9 (6.82)	4 (12.50)	
Ascites			
< 1000 mL	66 (50.00)	11 (34.38)	0.033
≥ 1000 mL	44 (33.33)	18 (56.25)	
Unintended ICU admission	12 (9.09)	8 (25.00)	0.014
Perioperative complication			
II–V n(%)	39 (29.54)	15 (46.87)	0.061
III–V n(%)	9 (6.82)	7 (21.87)	0.018*
Grade III + complication or unintended ICU admission n(%)	16 (12.12)	11 (34.37)	0.002
Intraoperative blood loss			
≤ 0.5L n(%)	66 (50.00)	20 (62.50)	0.442
0.5 ~ 1L n(%)	40 (30.30)	7 (21.87)	
≥ 1L n(%)	26 (19.69)	5 (15.62)	
Days to chemotherapy (median (IQR))	11 (9,19)	10 (9,27)	0.692
Cycle of chemotherapy			
No chemotherapy	15 (11.36)	5 (15.62)	0.747*
< 6 cycles n(%)	56 (42.42)	12 (37.5)	
≥ 6 cycles n(%)	60 (45.45)	13 (40.62)	
LOS (median (IQR))	20 (17,25)	19 (15,22)	0.323

IQR, interquartile range; CRS, *cytoreductive surgery;* PDS, primary debulking surgery (PDS); NACT/IDS, neoadjuvant chemotherapy followed by interval debulking surgery; SCS, Mayo Surgical Complexity Score; ASA, American Society of Anesthesiologist; ICU, intensive care unit; LOS, Hospital length of stay

^*^Fisher exact test

Out of the 58 patients who did not receive CRS, 51 discontinued treatment due to economic constraints or lack of will to be treated. Seven patients accepted laparoscopic/laparotomy exploratory surgery, the reasons why the surgery did not go ahead include: the family refused further surgery, or it was highly difficult to reach optimal cytoreductive surgery when taking into account the widespread area, or inadequate physical resilience of the patients. Compared with patients who have undergone CRS (Additional file 1: Table S2), the patients who did not receive surgery have worse ECOG (*p* < 0.001) and older age (*p* < 0.001). The rate of grade 2–3 comorbidities was higher in the nonoperative than in the operative group, but no significant difference was identified between the two groups (22% versus 15.9%, *p* = 0.324).

Table 4 presents the outcomes of univariable and multivariable regression analyses, investigating the association between ACE-27 and grade III + perioperative complication or unintended ICU admission. The primary events of Clavien–Dindo grade III–V complications or unintended ICU admission occurred in 27 patients (16.46%). On univariate analysis, ACE-27 score, SCS, albumin, CA125 at diagnosis, and ascites were statistically associated with grade III + complications or unintended ICU admission. On additional multivariable analysis, ACE-27 score (OR: 4.21; 95%CI 1.28–14.35; *p* = 0.018), albumin (OR: 4.82; 95%CI, 1.07–21.98; *p* = 0.038) and SCS (OR: 5.54; 95%CI, 1.72–19.86; *p* = 0.005) remained independently predictive of grade III + perioperative complication or unintended ICU admission.

**Table 4:** Univariate and multivariable logistic regression for severe 30-day postoperative complications or unintended ICU admission

Variants		Univariate analysis		Multivariable analysis	
		OR (95%CI)	*p*-value	OR (95%CI)	*p*-value
Age	< 70 years	ref			
	≥ 70 years	1.79 (0.75–4.18)	0.181		
Surgery type	NACT/IDS	ref			
	PDS	1.45 (0.55–4.6)	0.78		
ASA	I–II	ref			
	III–IV	1.59 (0.65–3.74)	0.295		
SCS	≤ 3	ref			
	≥ 4	3.2 (1.32–7.68)	**0.009**	5.54(1.72–19.86)	**0.005**
Operation time	≤ 4.5 h	ref			
	> 4.5 h	1.65 (0.72–3.86)	0.236		
Ascites	< 1000 mL	ref			
	≥ 1000 mL	2.53 (1.08–6.21)	**0.036**	2.09(0.71–6.38)	0.18
Albumin	≥ 30 g/L	ref			
	< 30 g/L	5.47 (1.55–19.53)	**0.007**	4.82(1.07–21.98)	**0.038**
CA125	< 600	ref			
	≥ 600	2.35 (0.95–6.42)	**0.077**	2.11(0.71–6.97)	0.195
BMI	< 25 kg/m^2^	ref			
	≥ 25 kg/m^2^	1.3 (0.52–3.09)	0.557		
FIGO stage	IIB	ref			
	III	3.4 (0.64–63.07)	0.248		
	IV	4.96 (0.78–97.19)	0.151		
ACE27	grade0-1	ref			
	grade2-3	3.8 (1.53–9.32)	**0.004**	4.21(1.28–14.35)	**0.018**
Pathological type	others	ref			
	serous	1.22 (0.42–4.44)	0.733		

OR, odds ratio; CI, confidence interval; ASA, American Society of Anesthesiologist; SCS, Mayo Surgical Complexity Score; BMI, body mass index; FIGO, Federation International of Gynecology and Obstetrics; ACE-27, Adult Comorbidity Evaluation 27

*P*-values of less than 0.1 in the univariate regression analysis were collected for the next step, and *p*-values of less than 0.05 were considered significant in further multivariate regression analysis

The predictive efficiency of elective variants (ACE-27, albumin, SCS) with Grade III + perioperative complications or unintended ICU admission was evaluated by plotting receiver operator characteristic (ROC) curves. Additional file 2: Fig. S1 shows the sensitivity, specificity, and area under the curve (AUC) for selected variables when used to predict the above short-term outcomes. The model was moderately accurate (0.7 < ​ AUC < ​ 0.8) in predicting whether old AEOC patients will ever suffer from Grade III + perioperative comorbidities or unintended ICU admission.

**Fig. 1:**
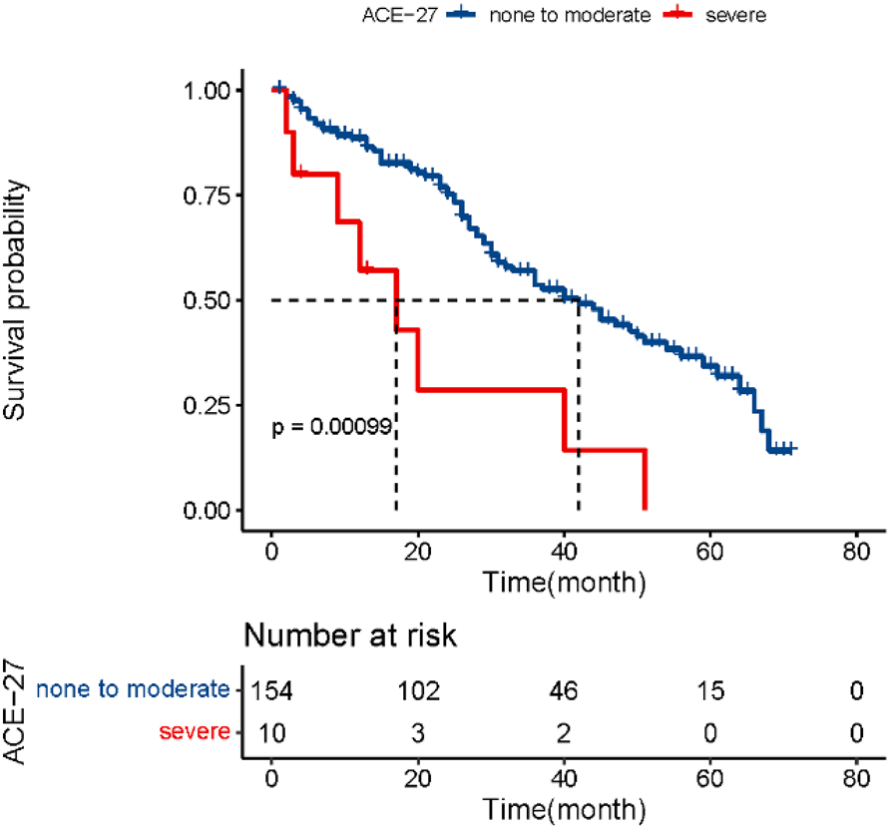
Relationship between ACE-27 and overall survival

In the end, Kaplan–Meier survival curves for overall survival (OS) were plotted and analyzed by log-rank test. The median survival time for overall survival was 43 months (range: 1–127 months). The result showed that the OS of patients graded severe was shorter than patients graded none to moderate (Fig. 1) (HR 3.25, 95%CI 1.55–6.79, *p* = 0.002). In multivariate analysis for OS controlling for ACE-27 and residual disease, grade severe by ACE-27 (HR 2.78, 95%CI 1.91–4.85, p = 0.031) and R2 of residual disease (HR 4.64, 95%CI 1.09–8.12, p = 0.023) were important predictors of OS. In addition, stratified analyses by age, BMI, FIGO stage, and pathology are presented in Additional file 1: Table S3 and Additional file 2: Fig. S2. For patients aged < 70 years, the OS of patients graded severe was shorter than patients graded none to moderate (*p* = 0.0004). The same conclusion can be obtained in the group of BMI < 25 kg/m^2^ (*p* = 0.001), the group of FIGO III stage (*p* = 0.021), and the group of pathology of serous (*p* = 0.016).

## Discussion

The accelerating aging population worldwide has been observed to augment the prevalence of older individuals with comorbidities, thereby underscoring the importance of thorough and multidimensional pre-operative assessments. Our recent study provides compelling evidence, demonstrating a robust correlation between the severity of comorbidities, as assessed by ACE-27, and post-surgical prognosis and overall survival. The higher grade of comorbidities rated by ACE-27 (moderate and severe) a patient suffers from, the more likely she is to develop serious perioperative complications or unintended ICU admission. The strength of the predictive capacity of ACE-27 for the studied perioperative outcomes is further corroborated by the area under the ROC curve, which reported values in excess of 0.70. Patients with severe complications also had shorter overall survival than those with none to moderate complications. This may provide clinicians with a fresh reference standard for clinicians to strengthen perioperative management.

The growing prevalence of multiple comorbidities among older people is a significant health concern [18], with approximately 35.26% of the individuals in our study manifesting at least two comorbidities. Comorbidities may be more common in cancer patients due to the enhanced medical surveillance these patients receive compared to their non-cancer counterparts [19]. Studies concerning the frailty index demonstrated that comorbidities as well as functional status, are associated with worse surgical outcomes and poorer OS [20, 21]. An array of studies have suggested an influential role of comorbidities in the process of ovarian cancer treatment [22, 23], though the incorporation more objective evaluative tools remains limited. In contrast to the classical comorbidity assessment tool CCI, which aggregates all items irrespective of severity, ACE-27 methodically categorizes comorbidities based on their severity. It was designed specifically for cancer patients and was determined after a thorough review of charts in medical records, rather than from the disease code. Importantly, the ACE-27 also takes into account the aspect of obesity. ACCI was not found to be associated with minor or major perioperative complications, as described in a previous study [7]. Our data showed that comorbidities of higher ACE-27 score (moderate and severe) significantly increase the risk for adverse outcomes after cytoreductive surgery in elderly women with AEOC. Existing data demonstrates a link between comorbidity and survival in ovarian cancer [22], however, contradictory evidence has also emerged with studies showing no such difference [8]. Notably, our study found a significantly inferior overall survival rate in cases with severe ACE-27 grades compared to those with none, mild, or moderate grades (p = 0.004).

Cytoreductive surgery, complemented by platinum and taxane-based chemotherapy, is presently the predominant therapeutic strategy for AEOC. Although older people who underwent surgery were confronted with a higher risk of perioperative death than young women [24], an increasing body of research corroborates its positive outcomes in older ovarian cancer patients. [25–27]. However, the adherence of elderly patients to operations is poor. This trend can also be attributed to the decision-making process of clinicians who often favor non-surgical procedures due to factors such as the patients' existing comorbidities and their perceived surgical resilience. The application of ACE-27 would provide an objective tool for clinicians in the process of making decisions. It must be clarified, however, that the usage of ACE-27 is not intended to exclude patients with moderate-to-severe comorbidities from surgery. Rather, it provides a useful tool to identify those patients necessitating intensive home support or multidisciplinary nursing teams during the perioperative period.

Several models have been developed to forecast complications within a 30-day and 90-day period following debulking surgery [28, 29]. A significant study that included 7029 patients suffering from ovarian cancer determined that Clavien–Dindo complications had a robust correlation with factors such as age, ASA, albumin, ascites, bleeding disorder, elective surgery, and procedure score, demonstrated through its strong internal validation with a C-index of 0.71 [30]. However, no model has included comorbidities, and indeed, they have not graded them. In our study, comorbidities, as graded by ACE-27 from moderate to severe, were highly predictive of adverse surgery outcomes, when controlling for risk factors including albumin, CA125, SCS, and ascites on multivariable analyses. This was further reinforced by the ROC curve that underscored the substantial predictive proficiency of the ACE-27 for the surgical outcomes investigated in our study. Consequently, predictive models incorporating graded comorbidities are expected to be developed in the future for more accurate prediction of survival and benefit from surgery.

The main goal of identifying risk factors for these surgical complications is to reduce their occurrence. In order to achieve this, a number of strategies have been adopted in our institution to minimize perioperative complications. Rigorous pre-operative assessment was implemented for patients with high ACE-27 grade comorbidities to reduce possible adverse outcomes. Regular weekly multidisciplinary meetings were conducted to achieve triage or specify rigorous surgical protocols for patients, and combined multidisciplinary surgery was performed when necessary. Enhanced recovery after surgery (ERAS) and venous thrombosis prevention strategies were also used to reduce the incidence of complications.

The findings from our research reinforce the significance of stratifying comorbidities in both clinical and research settings. Despite routine consideration in clinical decision-making, comorbidities have seldom been evaluated using objective, quantitative measures. Our study demonstrated for the first time that ACE-27 can be used to predict short-term surgical outcomes, as well as long-term outcomes in elderly individuals. The cohort in the study was limited to a typical group of newly diagnosed AEOC patients aged 65 years and above. We collected patient-centered and health resource outcomes essential to patients and their families, including grade III + perioperative complications and unintended ICU admission. ​Nevertheless, recognizably, there are several deficiencies in this study. First and most importantly, the sample size is relatively small, which necessitates that our findings need to be substantiated by larger, more extensive research. Secondly, while the ACE-27 would be an essential tool for risk stratification, it does not account for the full range of tolerability and treatment response to surgery. An integral clinical assessment remains necessary in eliciting other important perioperative factors that may influence both short-term and long-term outcomes.

## Conclusion

Overall, the ACE-27 offers a comprehensive, objective, and reliable tool for assessing comorbidity. It disaggregates severity of disease and suggests a potential for deployment on a wider clinical scale across a substantial patient population. We showed that higher score of ACE-27, independently of low albumin and high SCS, were highly related to severe perioperative complications or unintended ICU admission for elderly patients undergoing debulking surgery with AEOC. Equally noteworthy is the finding that the survival rate was considerably reduced among the population displaying severe ACE-27 grades compared to those with none to moderate scores. This underlines the tool's practical implications in patient management. By implementing ACE-27 early on, physicians can identify patients at greater risk, thereby tailoring specific interventions. In particular, such evaluations could improve surgical outcomes for ovarian cancer patients, supporting the selection of individuals needing perioperative rehabilitation. Moreover, it spurs a prevention-oriented approach, fostering collaborative action from the treatment team.

### Supplementary Information

**Additional file 1: Table S1.** univariate analysis between different comorbidities and adverse surgical outcomes. **Table S2.** Characteristics of patients received or not received CRS. **Table S3.** Log-rank test of OS for selected patients between group of none to moderate and of severe.

**Additional file 2: Fig. S1.** Receiver operator characteristic curve for Clavien–Dindo grade III–V complications or unintended ICU admission. **Fig. S2.** (a-d) Kaplan–Meier (K-M) survival curves of overall survival for selected patients. a. K-M survival curve for patients stratified by aged. b. K-M survival curve for patients stratified by BMI. c. K-M survival curve for patients stratified by FIGO stage. d. K-M survival curve for patients stratified by pathology.

## Data Availability

Not applicable.
