# Supplementary material for: Predictive value of the Adult Comorbidity Evaluation 27 on adverse surgical outcomes and survival in elderly with advanced epithelial ovarian cancer undergoing cytoreductive surgery
Source: Eur J Med Res. 2024 Mar 17;29:179. doi: 10.1186/s40001-024-01666-1 (PMC10946157; doi:10.1186/s40001-024-01666-1)
Supplement: Supplementary file 1 — Additional file 1: Table S1. univariate analysis between different comorbidities and adverse surgical outcomes. Table S2. Characteristics of patients received or not received CRS. Table S3. Log-rank test of OS for selected patients between group of none to moderate and of severe. [file 40001_2024_1666_MOESM1_ESM.doc]

Table S1 univariate analysis between different comorbidities and adverse surgical outcomes.

| Comorbidity | OR (95%CI) | *p* value |
| --- | --- | --- |
| Hypertension | 0.84 (0.36-1.91) | 0.672 |
| Diabetes mellitus | 0.86 (0.24-2.51) | 0.801 |
| Angina Coronary artery disease | 1.85 (0.56-5.34) | 0.278 |
| Respiratory system | 1.29 (0.19-5.53) | 0.756 |
| Congestive heart failure (CHF) | 17 (2.08-351.59) | **0.016** |
| Arrhythmias | 7.77 (1.61-41.64) | **0.01** |
| Venous disease | 2.32 (0.48-9.01) | 0.245 |
| Solid tumor | 1.49 (0.21-6.58) | 0.634 |
| Hepatic | 2.11 (0.29-10.42) | 0.387 |

Abbreviations: OR, odds ratio; CI, confidence interval;

Table S2 Characteristics of patients received or not received CRS.

| characteristic | no-CRS | CRS | *p* value |
| --- | --- | --- | --- |
|  | n=58 | n=164 |  |
| age (median (IQR)) | 73.7(69,78) | 68(66,70) | **0.000** |
| BMI (median (IQR)) | 21.78(19.53,24.65) | 22.88(20.50,25.55) | 0.101 |
| ECOG |  |  |  |
| 0 n(%) | 16(27.58) | 84(51.21) | **0.000** |
| 1 n(%) | 17(29.31) | 65(39.63) |  |
| ≥2 n(%) | 25(43.10) | 15(9.146) |  |
| FIGO |  |  |  |
| IIB n(%) | 3(5.172) | 18(10.97) | 0.194* |
| III n(%) | 24(41.37) | 114(69.51) |  |
| IV n(%) | 13(22.41) | 31(18.90) |  |
| Histology |  |  |  |
| serous n(%) | 37(63.79) | 136(82.92) | **0.010*** |
| mucinous | 4(6.896) | 9(5.487) |  |
| endometrioid | 4(6.896) | 5(3.048) |  |
| clear | 1(1.724) | 4(2.439) |  |
| Mixed+others n(%) | 12(20.68) | 10(6.097) |  |
| albumin |  |  |  |
| ＜30g/L n(%) | 12(20.68) | 12(7.317) | **0.009** |
| 30~35g/L n(%) | 10(17.24) | 32(19.51) |  |
| ＞35g/L n(%) | 19(32.75) | 78(47.56) |  |
| CA125 at diagnosis |  |  |  |
| ＜600 n(%) | 13(22.41) | 60(36.58) | **0.038** |
| ≥600 n(%) | 35(60.34) | 76(46.34) |  |
| ACE-27 |  |  |  |
| grade 0 n(%) | 20(34.48) | 49(29.88) | **0.013** |
| grade 1 n(%) | 19(32.75) | 83(50.61) |  |
| grade 2 n(%) | 8(13.79) | 22(13.42) |  |
| grade 3 n(%) | 11(18.96) | 10(6.10) |  |

Abbreviations: CRS, cytoreductive surgery; IQR, interquartile range; BMI, body mass index; ECOG, Eastern Cooperative Oncology Group; FIGO, Federation International of Gynecology and Obstetrics; ACE-27, Adult Comorbidity Evaluation 27;

* Fisher exact test.

Table S3 Log-rank test of OS for selected patients between group of none to moderate and of severe.

| Variants | | HR | 95%CI | *p* value |
| --- | --- | --- | --- | --- |
| age | ＜70 | 5.579 | 1.882-16.541 | **0.0004** |
|  | ≥70 | 2.298 | 0.790-6.686 | 0.11 |
| BMI | ＜25kg/m^2^ | 3.282 | 1.543-6.983 | **0.001** |
|  | ≥25kg/m^2^ | 0.048 | NA | 0.79 |
| FIGO | IIB | 16.087 | 1.006-257.199 | **0.0062** |
|  | III | 2.447 | 1.107-5.408 | **0.021** |
|  | IV | NA | NA | NA |
| pathology | serous | 2.915 | 1.165-2.292 | **0.016** |
|  | others | 1.951 | 0.532-7.156 | 0.29 |

Abbreviations: OS, overall survival; ACE-27, Adult Comorbidity Evaluation 27; HR, hazard ratio; CI, confidence interval; BMI, body mass index; FIGO, Federation International of Gynecology and Obstetrics;
