# Supplementary material for: Predictive value of the Adult Comorbidity Evaluation 27 on adverse surgical outcomes and survival in elderly with advanced epithelial ovarian cancer undergoing cytoreductive surgery
Source: Eur J Med Res. 2024 Mar 17;29:179. doi: 10.1186/s40001-024-01666-1 (PMC10946157; doi:10.1186/s40001-024-01666-1)
Supplement: Supplementary file 2 — Additional file 2: Fig. S1. Receiver operator characteristic curve for Clavien–Dindo grade III–V complications or unintended ICU admission. Fig. S2. (a-d) Kaplan–Meier (K-M) survival curves of overall survival for selected patients. a. K-M survival curve for patients stratified by aged. b. K-M survival curve for patients stratified by BMI. c. K-M survival curve for patients stratified by FIGO stage. d. K-M survival curve for patients stratified by pathology. [file 40001_2024_1666_MOESM2_ESM.doc]

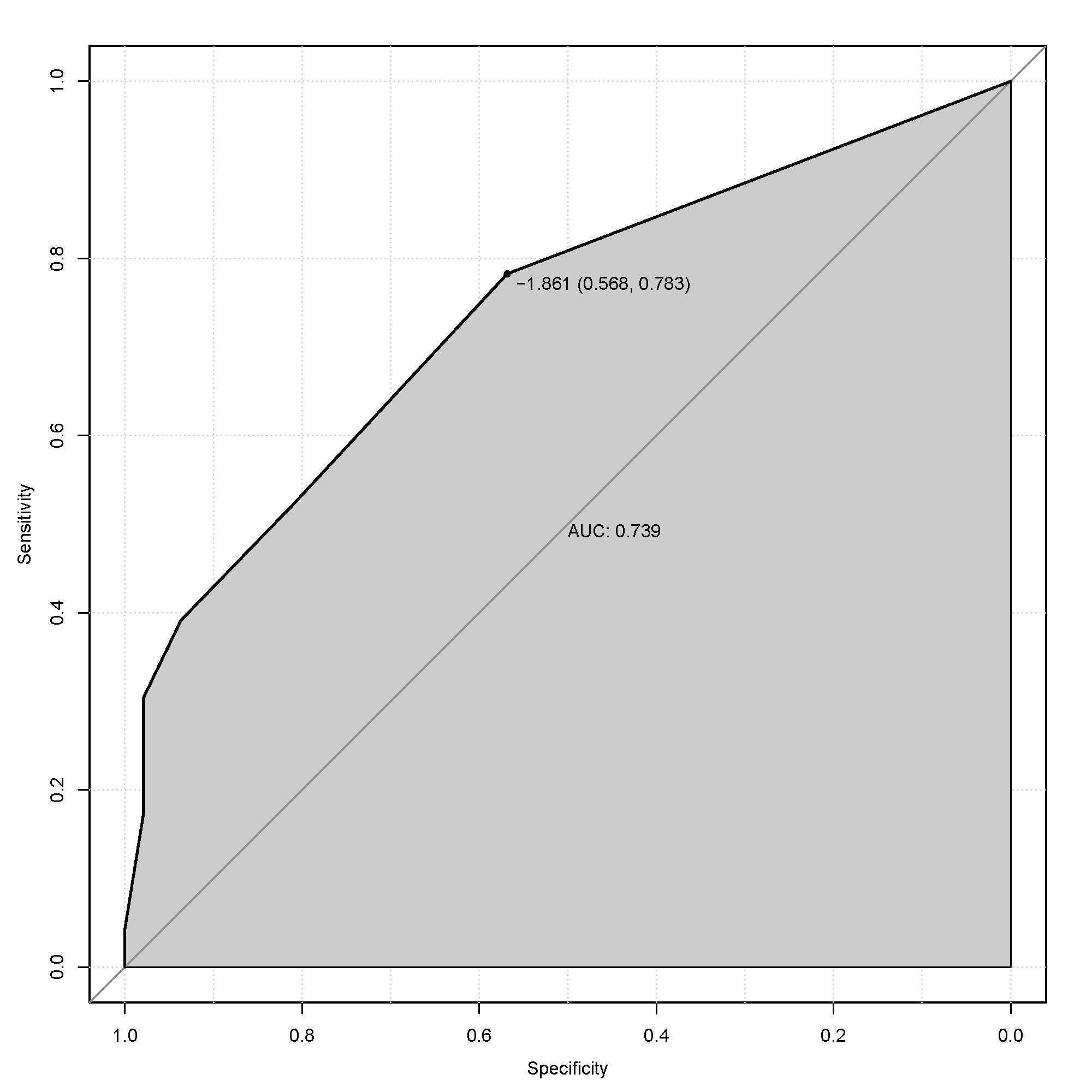


Fig. S1 Receiver operator characteristic curve for Clavien-Dindo grade III-V complications or unintended ICU admission.


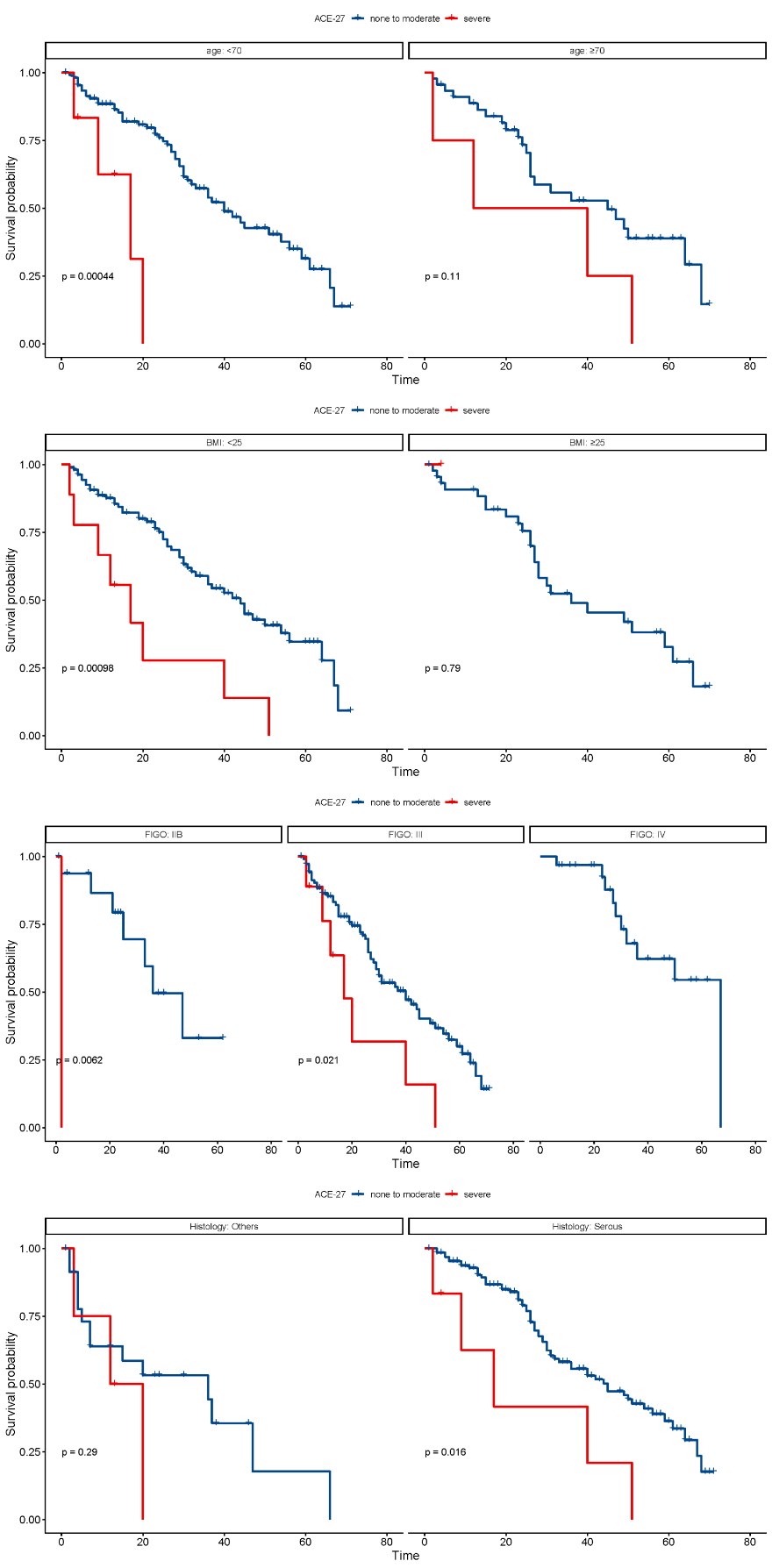


Fig. S2. (a-d) Kaplan-Meier (K-M) survival curves of overall survival for selected patients. a. K-M survival curve for patients stratified by aged. b. K-M survival curve for patients stratified by BMI. c. K-M survival curve for patients stratified by FIGO stage. d. K-M survival curve for patients stratified by pathology.
